# Ectopic Cushing Syndrome Due to a Large Mediastinal Neuroendocrine Tumor

**DOI:** 10.1210/jcemcr/luaf283

**Published:** 2026-02-27

**Authors:** Jorge Alberto Ramírez-García, Gabriela Garza-García, Roopa Mehta, Alfredo A Reza-Albarrán, Roberto De La Peña-López, Nicole M Iñiguez-Ariza

**Affiliations:** Department of Endocrinology and Metabolism, Instituto Nacional de Ciencias Médicas y Nutrición “Salvador Zubirán”, Mexico City 14080, Mexico; Department of Endocrinology and Metabolism, Instituto Nacional de Ciencias Médicas y Nutrición “Salvador Zubirán”, Mexico City 14080, Mexico; Department of Endocrinology and Metabolism, Instituto Nacional de Ciencias Médicas y Nutrición “Salvador Zubirán”, Mexico City 14080, Mexico; Department of Endocrinology and Metabolism, Instituto Nacional de Ciencias Médicas y Nutrición “Salvador Zubirán”, Mexico City 14080, Mexico; Department of Hematology and Oncology, Instituto Nacional de Ciencias Médicas y Nutrición “Salvador Zubirán”, Mexico City 14080, Mexico; Department of Endocrinology and Metabolism, Instituto Nacional de Ciencias Médicas y Nutrición “Salvador Zubirán”, Mexico City 14080, Mexico

**Keywords:** severe hypercortisolism, etomidate infusion, malignant tumor

## Abstract

Ectopic Cushing syndrome is an uncommon cause of hypercortisolism that needs rapid recognition and treatment to reduce complications. Here, we present the case of a 33-year-old man with a 1-year history of severe Cushing syndrome. Biochemical findings showed both high ACTH and 24-hour urine free cortisol, and nonsuppressed morning serum cortisol. The 18fluorine-1,4,7-triazacyclononane-1,4,7-triacetate-octreotide positron emission tomography/computed tomography revealed a large mediastinal tumor with high uptake. Initial biopsy reported a grade 1 neuroendocrine tumor with positive ACTH immunostaining. Treatment was initiated with ketoconazole and somatostatin analog. To achieve rapid Cushing syndrome control, etomidate intravenous infusion was started. After multidisciplinary assessment and because of high surgical risk (concern for airway compromise from tumor location above the trachea, size, and possible mechanical lung compression with laparoscopic adrenalectomy) debulking surgery of the primary tumor was performed first, followed by bilateral adrenalectomy. Pathology findings of the R2 tumor showed a higher grade tumor than previously reported and an 18 Fluorodeoxyglucose positron emission tomography/computed tomography demonstrated distant metastatic disease. In summary, severe hypercortisolism usually occurs in the setting of ectopic production, it can be debilitating with increased mortality, and in this case, tumor aggressiveness and location made management particularly challenging, requiring a multidisciplinary approach.

## Introduction

Ectopic Cushing syndrome (ECS) is a rare form of hypercortisolism resulting from an excess of ACTH, produced by extrapituitary tumor autonomous secretion [1]. ECS represents only 6% of all causes of endogenous Cushing syndrome (CS), stemming most frequently (2%) from a lung tumor, followed by mediastinal, pancreatic, and medullary thyroid neuroendocrine tumors [2].

Clinical manifestations of mediastinal tumors include those related to mass effect; however, the vast majority of ACTH-producing tumors cause hypercortisolism symptoms with up to two thirds of cases presenting with typical aggressive CS symptomatology [3].

In ACTH-dependent hypercortisolism, ACTH concentration will be inappropriately normal or elevated (SI: >20 ng/L). Imaging studies are useful to differentiate between Cushing disease and ECS [4]. The diagnosis of ECS is established when hypercortisolism is confirmed, and plasma ACTH is elevated from an extrapituitary source.

Although there is not a universal definition of severe CS, it has been proposed as an acute emergency if serum cortisol exceeds 36 μg/dL (SI: 1.296 nmol/L) at any time or when a 24-hour urinary free cortisol is over fourfold the upper limit and/or the occurrence of severe hypokalemia. Other characteristics that might suggest severe CS include: a recent onset of sepsis, opportunistic infections, uncontrolled hypertension, heart failure, gastrointestinal bleeding, myopathy, thromboembolism, and/or uncontrolled hyperglycemia [5].

Treatment for ECS includes surgery, radiation, and pharmacotherapy, aiming to normalize cortisol levels and reverse clinical features of the disease [6]. Although surgical tumor resection is the first line of treatment of ECS, prior pharmacotherapy is required to achieve hypercortisolism control, in an effort to decrease adverse events, though published evidence supporting this approach remains scarce [7].

Here, we describe the case of a young patient with severe hypercortisolism due to ECS, in whom management was particularly challenging owing to tumor location and size.

## Case Presentation

**Figure 1.**
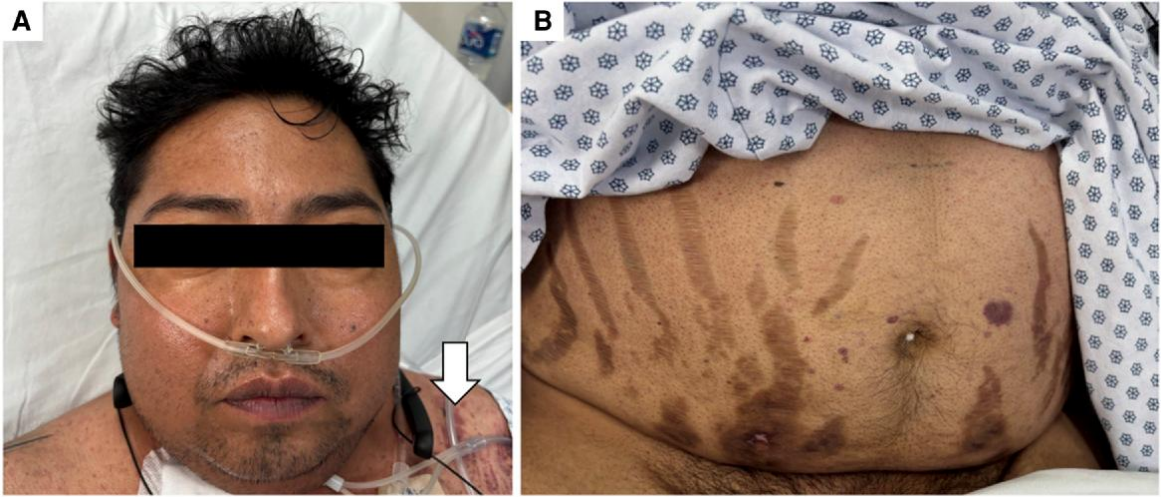
Patient characteristics at admission. (A) Patient with characteristic moon facies, facial plethora, and ecchymoses on the chest (white arrow). (B) Patient’s abdomen showing wide, hyperpigmented striae.

A 33-year-old man presented with a 1-year history of overt CS characterized by proximal myopathy, central obesity, and full-moon facies. He also developed purple skin striae and dorsocervical fat pad (Fig. 1). After 8 months, he developed progressive dyspnea and chest pain prompting medical assessment. During his initial evaluation, he had hypertension and a mediastinal mass on chest X-ray. Cortisol was high and therapy with ketoconazole 200 mg 3 times per day was initiated. He was referred to an oncologic center where biopsy showed a grade 1 well-differentiated neuroendocrine tumor, which led to his referral to endocrinology consultation within our hospital.

Upon admission, the vital signs were mostly in normal range with blood pressure of 124/62 mm Hg, heart rate 82 beats per minute, respiratory rate of 19 breaths per minute, and oxygenation of 86%. Physical examination revealed the stigmata of CS previously described, along with basal pulmonary crackles and hypoxia.

## Diagnostic Assessment

**Figure 2.**
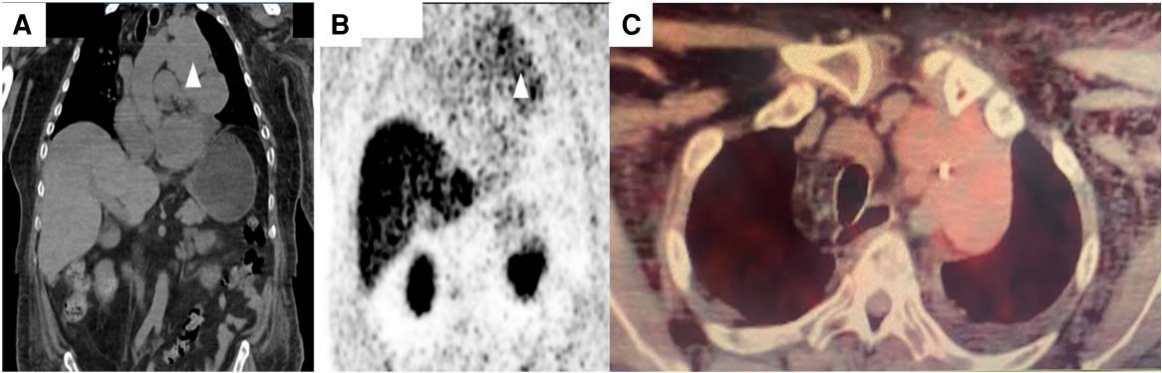
(A) Contrast-enhanced CT image showing a 13.5 × 6.4 cm mediastinal tumor (white arrow). (B) Scintigraphy demonstrating physiologic uptake of 18F-NOTA-octreotide in the liver and adrenal glands, and pathologic uptake in the mediastinal tumor (white arrow). (C) Axial fused PET/CT with 18F-NOTA-octreotide showing heterogeneous uptake in the mediastinal tumor with an SUVmax of 3.1.

Laboratory tests at admission are shown in Table 1. A pituitary magnetic resonance imaging scan was performed before referral, showing no sellar mass. To further characterize the possible ectopic tumor origin in the setting of a neuroendocrine mediastinal tumor, a 18 fluorine-1,4,7-triazacyclononane-1,4,7-triacetate (18F-NOTA)-octreotide positron emission tomography (PET)/computed tomography (CT) scan was conducted, revealing adenopathy and a multilobulated mediastinal tumor surrounding the left brachiocephalic trunk, measuring 13.5 × 6.4 cm, with mild focal and heterogeneous uptake, with a standard uptake value (SUV) of 3.1 (Fig. 2).

**Table 1.** Biochemical findings at admission and after adrenalectomy

	Admission	After adrenalectomy	Reference range
**Morning serum cortisol**	50 µg/dL (1382 nmol/L)	2.85 µg/dL (78.66 nmol/L)	6.7-23 µg/dL (185-624 nmol/L)
**ACTH**	316 pg/mL (316 ng/L)	380 pg/mL (380 ng/L)	10-50 pg/mL (10-50 ng/L)
**Urinary free cortisol**	2243 µg/24 hours (6191 nmol/24 hours)		58-403 µg/24 hours (160-1112 nmol/24 hours)
**Overnight low-dose dexamethasone suppression test**	60 µg/dL (1656 nmol/L)		<1.8 µg/dL (<0.06 nmol/L)

Histopathological examination of the mediastinal mass confirmed a grade 1 neuroendocrine tumor, positive for chromogranin, synaptophysin, and ACTH (20%) with a Ki-67 proliferation index of 1%. Based on these findings, a diagnosis of ectopic CS was established.

## Treatment

**Figure 3.**
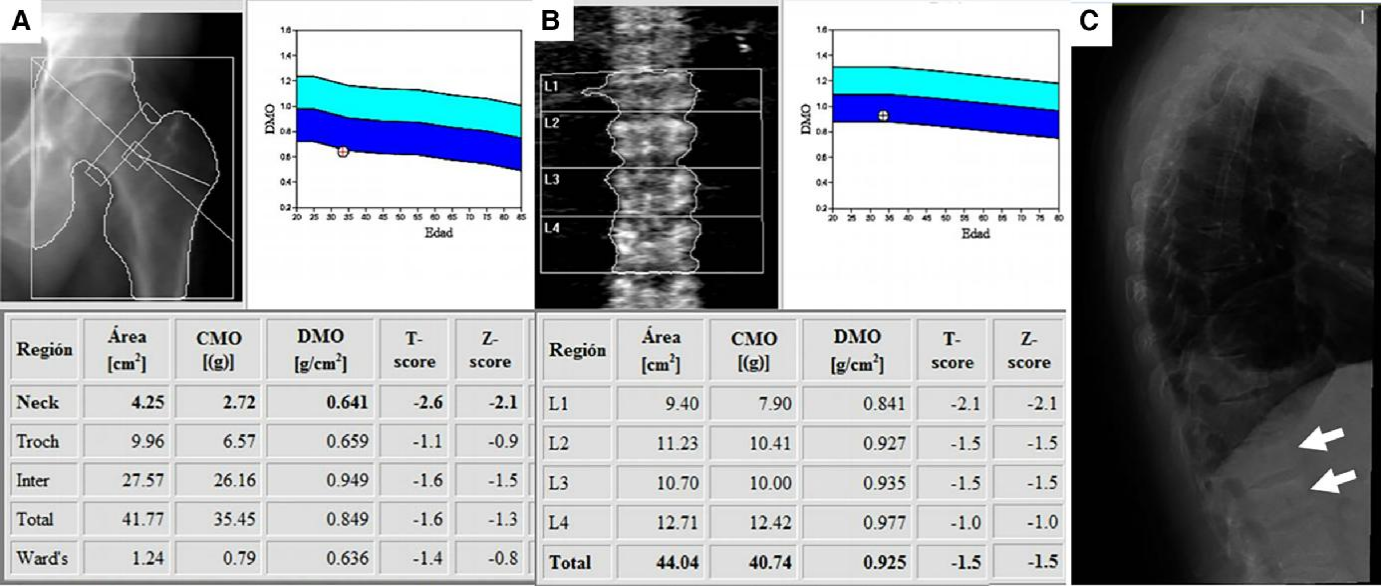
(A) Dual-energy X-ray absorptiometry (DXA) scan of the femoral neck and total hip showing low bone mineral density. (B) DXA scan of the lumbar spine showing low bone mineral density. (C) Lateral X-ray of the lumbar spine showing diminished vertebral body height with wedge deformity, indicative of vertebral fractures (white arrows).

Abbreviations: CMO, Bone Mineral Content; DMO, Bone Mineral Density.

Because of the patient's severe symptoms, treatment was initiated promptly with ketoconazole, titrated up to a maximum dose of 1200 mg per day, alongside with octreotide long-acting release 20 mg every 21 days. Despite treatment, serum cortisol and ACTH concentrations remained elevated, with complications of hypercortisolism such as hospital-acquired pneumonia requiring oxygen therapy, subsegmental pulmonary embolism shown on CT scan at admission, hypertension, diabetes, and proximal weakness that refrained him from walking, *Malassezia furfur* folliculitis and severe osteoporosis with multiple compression fractures of the lumbar spine and bone mineral density T-scores of: -1.5 lumbar spine, -2.6 femoral neck, and -1.6 total hip (Fig. 3).

To achieve rapid control, an etomidate intravenous infusion was started at an initial dose of 0.01 mg/kg/h, increased to a maximum of 0.04 mg/kg/h, which was well tolerated. While cortisol concentration decreased during etomidate infusion, long-term use was not feasible.

**Figure 4.**
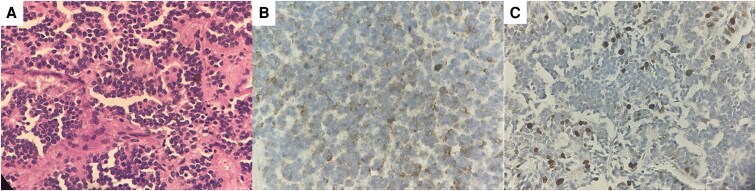
(A) Hematoxylin-and-eosin stain of a 40 × image showing a neoplasm of round, blue cells arranged in lobules with salt and pepper chromatin. (B) Tissue with positive focal chromogranin. (C) Ki-67 proliferation index of 10%.

We considered a bilateral adrenalectomy upfront to prioritize resolution of severe hypercortisolism. However, during preoperative evaluation, the anesthesia team considered it too risky to operate on the adrenals because of the mediastinal tumor location and size, which could compromise airway permeability upon sedation, in addition to the required position during surgery and the need of pneumoperitoneum that could decrease cardiac output and mechanically compress the lungs, considering it an even higher risk surgery with an increased mortality potential. After multidisciplinary consensus weighing all risks and benefits, debulking surgery of the primary tumor was elected first, and thus, an R2 resection of the mediastinal tumor was performed. Histopathological examination revealed a grade 2 neuroendocrine tumor (Ki-67: 10%) with lymphovascular invasion, positive lymph nodes, and positive margins (Fig. 4).

**Figure 5.**
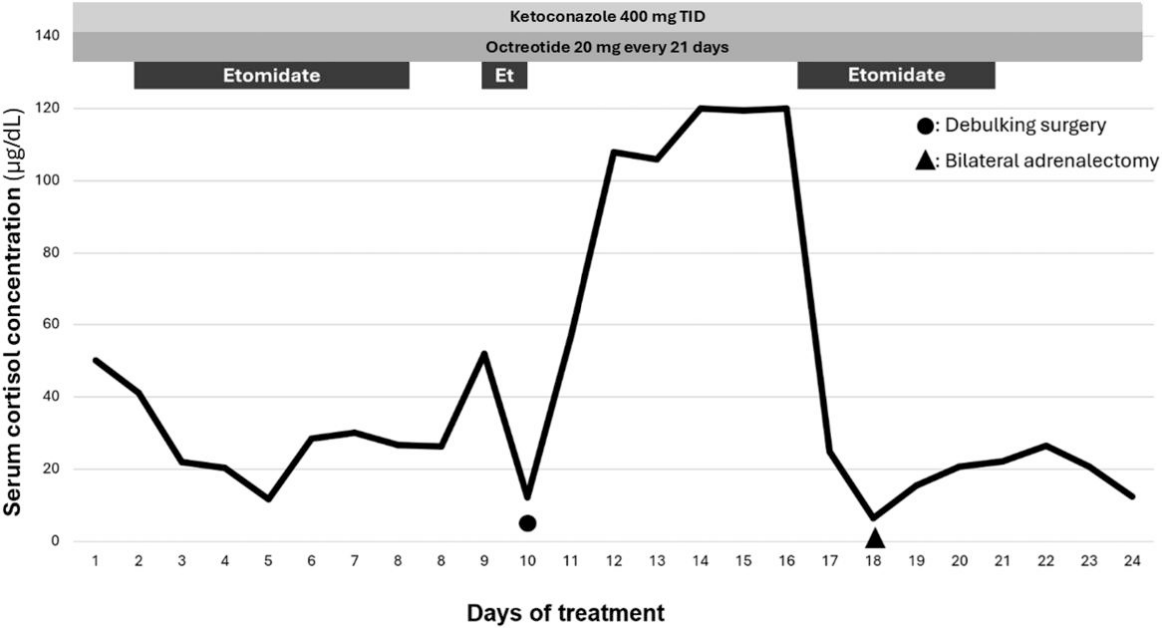
Management timeline and serum cortisol concentration. Abbreviation: Et, etomidate.

Following debulking of the mediastinal tumor, hypercortisolism persisted, and etomidate infusion was restarted; once serum cortisol was reduced to below 20 µg/dL (SI: 552 nmol/L), bilateral adrenalectomy was performed (Fig. 5), and on pathology adrenal hyperplasia was described.

## Outcome and Follow-up

Following adrenalectomy, serum cortisol concentrations normalized, and the patient was discharged on hydrocortisone and fludrocortisone replacement. At a 1-month follow-up, the patient showed significant improvement, with no orthostatic symptoms and remission of both diabetes and hypertension. However, hyperpigmentation was noted; laboratory tests are shown in Table 1 and chromogranin A of 196.60 ng/mL was noted (SI: 196.60 µg/L) (reference range: <100 ng/mL; SI: <100 µg/L).

**Figure 6.**
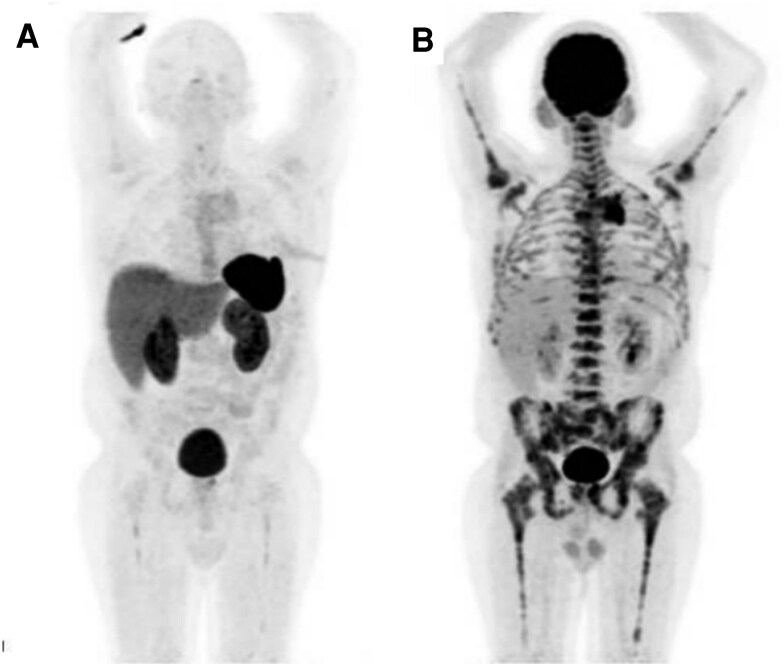
(A) 18F-NOTA-octreotide PET/CT with low uptake in the remaining tissue. (B) 18FDG PET/CT with increased uptake in the mediastinal lesion, mediastinal lymph nodes and bone lesions.

A follow-up 18F-NOTA-octreotide PET/CT scan revealed low uptake in the remaining tissue (Fig. 6A), whereas an 18-fluorodeoxyglucose (18F-FDG) PET/CT demonstrated increased uptake in the mediastinal lesion, as well as in mediastinal lymph nodes and bone lesions (Fig. 6B).

Considering these findings, the patient was started on palliative radiotherapy to lumbar bone lesions and cytotoxic chemotherapy with cisplatin and etoposide. However, after 4 cycles he had progressive disease. Thus, second-line chemotherapy with oxaliplatin and capecitabine was started. Unfortunately, tumor response has been poor, with high uptake in the mediastinal lesion on a follow-up 18F-FDG PET/CT and increase in serum ACTH to 1000 pg/mL (SI: 1000 ng/L).

## Discussion

Endogenous hypercortisolism caused by a mediastinal tumor is rare, accounting for only 0.5% of cases. Furthermore, primary neuroendocrine tumors (NETs) of the mediastinum represent approximately 2% to 4% of all mediastinal tumors [8]. Symptoms associated with these tumors vary depending on their localization; most commonly, they include cough (60%), chest pain (30%), fever (20%), and dyspnea (16%). However, systemic signs and symptoms related to paraneoplastic activity may also occur, including myasthenia gravis, hypercalcemia, hypokalemia, thyrotoxicosis, and CS, among others [9].

Given that CS is a rare and potentially life-threatening condition if not managed appropriately, prompt recognition is crucial. Studies have shown that, on average, the diagnosis of CS is delayed by approximately 34 months. However, in cases like ours, where disease manifests aggressively, the diagnosis can be reached more rapidly [10]. Upon admission, the patient presented with overt and aggressive features of CS, making the diagnosis evident.

Although an 8-mg dexamethasone suppression test was not performed during the diagnostic evaluation before referral to our institution, the identification of a hypermetabolic tumor in the mediastinum through imaging, along with histopathological findings, allowed for the establishment of an appropriate diagnosis. We opted not to pursue a high-dose dexamethasone test in this case, because he already had a negative pituitary magnetic resonance imaging scan and a mediastinal tumor biopsy with positive ACTH immunostaining. However, despite its limitations including an 81% and 66.7% sensitivity and specificity for diagnosing pituitary-dependent CS respectively, we acknowledge the importance of performing a high-dose dexamethasone suppression test during assessment of CS [11].

The primary goal of ECS treatment is to normalize cortisol concentration to resolve signs, symptoms, and comorbidities associated with hypercortisolism. Although complete surgical resection of the ACTH-producing tissue is first-line therapy, prior pharmacotherapy is required to reduce cortisol before any surgical procedure [6]. In this case, despite initiating management with dual therapy, hypercortisolism persisted. Consequently, treatment with etomidate was initiated due to its potent inhibition of 11β-hydroxylase, which catalyzes conversion of 11-deoxycortisol to cortisol. Etomidate offers advantages such as parenteral administration and rapid onset of action. Although some infusion-related side effects have been reported, none was observed. In our experience, we have used etomidate outside of the intensive care unit with lower doses as reported in the literature [12].

Bilateral adrenalectomy is recommended for patients with active disease when all other treatment options have been suboptimal or have failed. In our case, the procedure was performed due to the severity of the clinical and biochemical behavior of CS, and the impossibility to achieve an R0 resection of the tumor. However, it is important to emphasize that this is a high-risk procedure, particularly in the context of our patient. Notably, ectopic ACTH secretion is associated with a high mortality rate, with a median mortality of 17% at 41 months of follow-up, and a disproportionately high mortality of approximately 40% within the first year. Additionally, residual cortisol secretion may occur in up to 34% of patients, although recurrent hypercortisolism is observed in less than 2% of cases.

The case here was particularly challenging because of the location of the mediastinal tumor. Initially, when adrenalectomy was proposed, the procedure was classified as high risk given the presence of severe postural symptoms and tracheobronchial compression greater than 50% related to the mediastinal mass [13]. Not only the procedure itself increased the risk of mortality but mediastinal masses have been associated with high mortality, particularly in patients with massive effusion, pneumonia, and respiratory failure [14], in which only pneumonia was present in our patient.

Although patients may continue to experience fatigue after adrenalectomy, an overall improvement in quality of life is typically observed after CS control, as documented in our patient, who is now able to walk, and transitioned from wheelchair to cane to walking without aid. It is important to highlight that ECS is associated with the poorest prognosis [15]. Predictors of prognosis in malignant NETs of the thymus like size (bigger than 5-7 cm), sex, and age have been studied; however, these remain controversial. The overall 5-year survival of malignant NETs of the thymus varies from 20% to 80%, with a median survival of 60 months [16].

Finally, we acknowledge the existence of novel treatments that could be considered as adjunctive therapies alongside chemotherapy, such as Lutetium-177-Dotatate, which has demonstrated efficacy in the management of midgut NETs by significantly prolonging progression-free survival [17]. However, its effectiveness in other types of NETs, such as mediastinal tumors, remains obscure.

In conclusion, this case highlights the atypical presentation of ectopic CS resulting from a mediastinal NET. Treatment approach was particularly challenging because of the location and size of the ACTH-producing tumor, the aggressiveness of the patient’s hypercortisolism, and the lack of response to initial therapy. In patients with aggressive CS, it is crucial to establish prompt management to reduce the risk of comorbidities and mortality.

## Learning Points

- Ectopic Cushing syndrome can be particularly aggressive because of autonomous excessive production of ACTH, which, if not managed appropriately, can result in high morbidity, significant reduction in quality of life, and, in worse case scenarios, death.
- Management of severe hypercortisolism resulting from ectopic Cushing syndrome typically involves both pharmacological and surgical therapies, and a definitive approach to hypercortisolism should be established. This case highlights that tumor location can further complicate surgical management.
- Malignant metastatic neuroendocrine tumors causing ectopic Cushing syndrome are particularly aggressive.
- Follow-up care for all patients with a history of endogenous hypercortisolism is essential because of the risk of complications related to adrenal insufficiency and, less commonly, residual cortisol secretion.
- Response to etomidate infusion is fast after initiation, achieving rapid control on hypercortisolism.

## Data Availability

Data sharing is not applicable to this article as no datasets were generated or analyzed during the current study.
